# The Components of Bone and What They Can Teach Us about Regeneration

**DOI:** 10.3390/ma11010014

**Published:** 2017-12-22

**Authors:** Bach Quang Le, Victor Nurcombe, Simon McKenzie Cool, Clemens A. van Blitterswijk, Jan de Boer, Vanessa Lydia Simone LaPointe

**Affiliations:** 1 Institute of Medical Biology, Agency for Science, Technology and Research (A*STAR), 8A Biomedical Grove, #6-06 Immunos, Singapore 138648, Singapore; lequangbach@gmail.com (B.Q.L.); victor.nurcombe@imb.a-star.edu.sg (V.N.); simon.cool@imb.a-star.edu.sg (S.M.C.); 2 Department of Orthopaedic Surgery, Yong Loo Lin School of Medicine, National University of Singapore, NUHS Tower Block, Level 11, 1E Kent Ridge Road, Singapore 119288, Singapore; 3 Department of Complex Tissue Regeneration, MERLN Institute for Technology-Inspired Regenerative Medicine, Maastricht University, P.O. Box 616, 6200 MD Maastricht, The Netherlands; c.vanblitterswijk@maastrichtuniversity.nl; 4 Department of Cell Biology-Inspired Tissue Engineering, MERLN Institute for Technology-Inspired Regenerative Medicine, Maastricht University, P.O. Box 616, 6200 MD Maastricht, The Netherlands; jan.deboer@maastrichtuniversity.nl

**Keywords:** bone healing, fracture healing, bone tissue engineering, bone anatomy

## Abstract

The problem of bone regeneration has engaged both physicians and scientists since the beginning of medicine. Not only can bone heal itself following most injuries, but when it does, the regenerated tissue is often indistinguishable from healthy bone. Problems arise, however, when bone does not heal properly, or when new tissue is needed, such as when two vertebrae are required to fuse to stabilize adjacent spine segments. Despite centuries of research, such procedures still require improved therapeutic methods to be devised. Autologous bone harvesting and grafting is currently still the accepted benchmark, despite drawbacks for clinicians and patients that include limited amounts, donor site morbidity, and variable quality. The necessity for an alternative to this “gold standard” has given rise to a bone-graft and substitute industry, with its central conundrum: what is the best way to regenerate bone? In this review, we dissect bone anatomy to summarize our current understanding of its constituents. We then look at how various components have been employed to improve bone regeneration. Evolving strategies for bone regeneration are then considered.

## 1. Introduction

The human skeletal system consists of 206 bones and provides a rigid support for every other organ in the body. Some organs, like the brain and spinal cord, are protected inside bone structures—the skull and vertebrae, respectively. Other organs, such as the muscles, are attached to the skeleton. The skeleton is an adaptive structure, and as it grows through childhood, and the rest of the body grows along with it. Apart from providing structure and protection, the skeletal system functions to cooperate with joints and muscles for movement. Other critical functions of the skeletal system include blood cell production, mineral storage, and endocrine regulation [1].

Throughout life, the skeletal system has to endure great physical stress, predisposing it to injuries and disorders. Fortunately, the body has developed superb mechanisms to adapt and regenerate bone. For example, bone strength can increase in response to weight gain or following athletic training [2], and with minimal clinical intervention, fractured bones can heal into functionally normal bone [3]. Unfortunately, in 5–10% of cases where healing is compromised, the economic and health burden is significant. The Global Burden of Disease study (2013) found musculoskeletal conditions, such as arthritis and back pain, affect more than 1.7 billion people worldwide. These conditions are the leading cause of years lived with disability in 86 countries, and the second or third leading cause in 67 countries [4].

Three major breakthroughs have fueled recent advances in bone research. First, the discovery of bone morphogenetic proteins (BMPs) in 1965 initiated a new era of research and development for bone growth factor therapy [5]. The discovery of mesenchymal stem cells (MSCs) in 1991, coincident with the first isolation of human embryonic stem cells, also stimulated significant interest [6]. Finally, the development of materials mimicking bone extracellular matrix, including calcium phosphate ceramics, collagens, and glycosaminoglycans, exponentially increased the number of available alternatives to bone graft [7]. With this, the concept of a tissue engineering “triangle” consisting of growth factors, cells, and scaffolds has continued to provide a growing list of bone graft substitutes. 

For those trying to develop bone graft substitutes through biomimicry, it is important to thoroughly and diligently study bone and its constituents. In this review, we consider the most current and salient facts pertaining to skeletal healing. We review the constituents of bone and the contributions each component makes to bone healing. Additionally, we ask, what has been tested and what does and does not work when used for treating bone injuries?

## 2. Dissecting Bone at the Tissue Level

### 2.1. Periosteum

**Figure 1:**
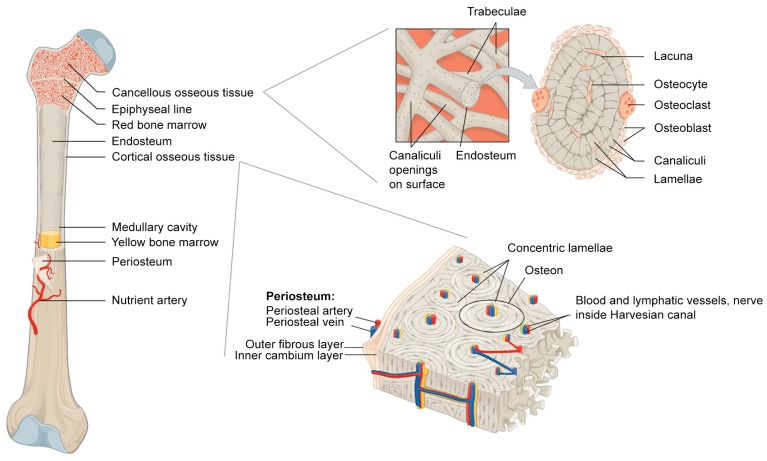
Bone anatomy. Modified and combined from Wikimedia Commons by OpenStar College CC BY 3.0 license [11].

The outermost layer of almost every bone in the body comprises the periosteum, a dense bilayer membrane responsible for appositional bone growth in children, partial blood supply to bone, and bone fracture repair (Figure 1). Periosteum consists of two distinct layers, an outer fibrous layer providing structural support, and an inner cambium layer abundant in osteoprogenitor cells. As early as the 18th century, surgeons discovered that periosteum itself had the potential to induce new bone formation [8,9]. Today, surgeons take care not to disturb or remove the periosteum around sites of injury as they believe it is one of the most crucial components of bone healing [10]. Despite this important function, periosteum receives less attention from scientists than other bone components.

The idea of utilizing a periosteal graft to augment bone healing at distant sites is not novel, but the problem with transplanting the periosteum is that its blood supply must be maintained to keep the osteogenic cells viable. In the 1990s, Doi and Sakai demonstrated the use of vascularized periosteal flaps, consisting of a thin flap of periosteum with its intact blood supply, to cover bone defects [12]. This method has been used successfully to treat difficult cases of large bone defects and persistent pseudarthrosis [13,14,15]. An interesting experiment by Saito et al. tested the osteogenic capacity of vascularized periosteum by wrapping it around beta-tricalcium phosphate (b-TCP) before implantation in rabbit muscle [16]. Bone formation was observed in the group with vascularized periosteum, but incomplete osteogenesis was found if the periosteum was non-vascularized, and no bone was found if b-TCP alone was implanted. This experiment emphasized the importance of a blood supply to the periosteal graft to successfully direct bone repair in vivo. 

In addition to the need for vascularization, another issue with periosteum is the thickness of its inner cambium osteoprogenitor layer decreases with age, concomitant with a decrease in its osteogenic potential [17]. The cells responsible for the osteogenic potential have been shown to be MSCs, and despite being more difficult to harvest, periosteal MSCs have been shown to be a better healing source than MSCs harvested from bone marrow [18,19]. Intriguingly, MSCs from periosteum possess unique chondrogenic potential, as demonstrated by the major contribution they make to the cartilaginous callus during fracture healing. Indeed, removal of the periosteum, but not the bone marrow or endosteum, inhibits cartilage formation and endochondral ossification [20]. In addition to the decreased osteogenic capacity with ageing, the anatomic location of the cells appears to be important. Studies have shown that tibial periosteum has more osteogenic potential than that of the calvaria [21,22].

As with many tissues, the limited availability of periosteum hinders its use as an autograft in multiple or large bone defects. As such, attempts have been made to engineer constructs that mimic periosteal functions for bone regeneration [23,24,25,26]. Baldwin et al. recently created an orthotopic xenograft model to evaluate their tissue-engineered periosteum, a construct that included osteoprogenitor and vascular compartments [27]. Their multiphasic construct combined a star-polyethylene glycol (PEG) heparin hydrogel system loaded with human umbilical vein endothelial cells (HUVECs) with a poly (ε-caprolactone) tubular scaffold seeded with human bone marrow-derived MSCs. After 30 days in vivo, the transplanted MSCs in the hydrogel layer retained their undifferentiated phenotype and the HUVECs developed into mature functional vessels connected to the host vasculature. Their results confirmed that the periosteum is a promising tissue that can be used to regenerate bone effectively if and only if it is well-vascularized.

### 2.2. Osseous Tissue

Osseous tissue is the rigid mineralized tissue that accounts for the structural aspect of skeletal function, and can, therefore, be said to define bone. Formally, osseous tissue is that which is enveloped between the periosteum and the endosteum, excluding the bone marrow. Previously, it was thought that osseous tissue lacked osteoprogenitor cells and that only osteocytes were present, apart from vascular cells inside Haversian canals [28]. Recently, MSCs have been isolated from osseous tissue using collagenase digestion and have been demonstrated to be essentially equivalent to bone marrow-derived MSCs [29,30]. 

Osseous tissue of cortical bone and cancellous bone are biochemically identical, but structurally distinct. It consists of an organic phase of mainly collagen fibres, which impart strength, flexibility, and resistance to torsional force, and an inorganic phase of mainly hydroxyapatite, which provides resistance to compression. Structurally, cortical osseous tissue can be described as a solid containing a series of voids (Haversian canals, Volkmann’s canals, lacunae, and canaliculi) with an overall porosity of ~10%. Cancellous osseous tissue, on the other hand, is a network of small, interconnected plates of trabeculae with relatively large spaces between them, having a porosity between 50% and 90% [31]. Osseous tissue is the primary component in many bone graft materials, including autogenous, allogeneic (fresh frozen, freeze-dried, and/or demineralized), and xenogeneic (irradiated, deproteinized, and/or demineralized) bone grafts. However, to isolate the effect of osseous tissue from other tissues, one would need to use either cortical bone without the periosteum or processed cancellous bone that does not contain bone marrow. Under such conditions it has been realized that osseous tissue alone is not only a good osteoconductive material, it also possesses slightly osteoinductive activity, especially when used fresh [32]. In the field of bone regeneration, osteoconductive refers to the property of the grafting material to guide the natural reparative growth of bone, while osteoinductive refers to the ability to induce bone formation by recruiting osteoprogenitor cells and stimulating osteogenesis [33]. 

Cortical bone contains significantly more osseous tissue per unit weight than cancellous bone, and, thus, is better at providing the initial mechanical stability and strength for bony defects. However, the structure of this dense cortical osseous tissue becomes a barrier to vascular ingrowth and remodelling [34,35]. An exception to this is the autologous vascularized cortical (e.g., fibular) bone graft that has both superior mechanical properties and speed of healing [36]. Cancellous bone provides little initial mechanical strength, but its porous structure allows rapid invasion by blood vessels and, hence, faster remodelling. In practice, physicians control the balance between initial mechanical strength and remodelling/healing speed by adjusting the ratio of cortical/cancellous bone they use [37]. 

The osteoconductive properties of osseous tissue have been leveraged to create scaffolds that maximize bone remodelling capacity. Bone cells such as osteoclasts, macrophages and osteoblasts naturally form basic multicellular units on the surface of osseous tissue, following specific remodelling signals to repair structural damage or respond to changes in mechanical loading [38]. Without vasculature, an osseous tissue graft quickly becomes necrotic, such that the whole graft must be remodelled—resorbed and replaced with new osseous tissue—for healing to be completed [39]. The mineralized matrix of osseous tissue also harbours abundant growth factors including IGFs and BMPs that have mitogenic and osteogenic activity [40], suggesting that osseous tissue is also osteoinductive. The benefit of this growth factor reservoir depends on how the osseous tissue is processed, because this affects growth factor stability, availability, and bioactivity. Autologous osseous tissue is often minimally processed and used fresh, thus maintaining its full osteoinductive potential. Allogeneic and xenogeneic osseous tissue in contrast must be processed to eliminate the immunogenicity that causes graft rejection. Demineralized bone matrix (DBM) is osseous tissue processed to remove all cells and almost all minerals; what remains is an organic extracellular matrix that has been shown to be abundant in growth factors [41]. Nevertheless, it is not easy to process osseous tissue while preserving the endogenous growth factors. Different methods combined with variable starting materials results in inconsistent end products [42]. Despite the number of versions on the market, the efficacy of DBM for bone healing has recently been questioned [43,44]. However, taken together, osseous tissue is an excellent scaffold for bone regeneration, though its full osteogenic potential depends on the source of the tissue and the processing methods. Osseous tissue has been, and is currently used in combination with other bone components, such as cells and bone growth factors, to achieve better overall grafting performance.

### 2.3. Endosteum

Endosteum is the least studied component of bone. A PubMed search for titles containing “endosteum” or “endosteal” surprisingly only yields 446 results at the time of writing; this compares to 2559 for “periosteum” or “periosteal”, 6091 for “osseous”, and 63,165 for “bone marrow” (Table 1). Endosteum arises from periosteum, which becomes entrapped during appositional bone growth. As periosteal osteoblasts form new bone matrix on the bone surface, part of the periosteum and its vasculature is slowly engulfed in newly-formed osteons. The blood vessels become the Haversian blood vessels that nurture osseous tissue, while the periosteum becomes the endosteum lining Haverisian canals and medullary cavities [45]. Unlike the periosteum, the endosteum is a very thin membrane, averaging only 10–40 µm in thickness, consisting of an indistinct connective tissue layer and a few layers of cells [46]. The cells in the endosteum are arranged in a mosaic pattern of formative, resting, and resorptive areas, characterized by the presence of active osteoblasts, preosteoblasts or osteoclasts, respectively [47]. Functionally, endosteum contributes to bone repair and reconstruction just as much as the periosteum, as it houses osteoprogenitor cells such as MSCs and preosteoblasts [48]. The endosteum also serves to control the weight-to-strength ratio of long bones by resorbing unnecessary osseous tissue from the medullary cavity as bones are growing in thickness. Due to its thin and indistinct appearance, endosteum as a tissue is less favoured by orthopaedic surgeons than other bony components. As endosteum originates from periosteum, it would appear to be more logical to use the periosteum, which is thicker, easier to handle, replete with its own blood vessels, and contains more osteoprogenitor cells. The resorptive capability of endosteum may be more useful in controlling bone overgrowth or even undesirable ectopic bone formation. However, this ability, largely uncharacterized and poorly understood, is the result of careful adjustments of the bone remodelling signalling system, tilting the balance toward controlled bone resorption. In short, the endosteum, on its own, has not been recognized as a suitable source for tissue engineering of grafts for bone healing and regeneration. Despite that, the complex molecular mechanism resulting in the bone resorptive property of the endosteum clearly warrants further investigation.

### 2.4. Bone Marrow

Bone marrow is one of the vulnerable soft tissues that is protected inside bone. Despite being part of bone anatomy, bone marrow is better characterized by its function as a blood organ. Of the 63,165 hits on PubMed with “bone marrow” in the title (the search term not including “mesenchymal stem cells”), only 805 hits can be found to couple with either “bone regeneration”, “bone healing”, “bone tissue engineering”, “fracture healing”, or “non-union” in the title/abstract, accounting for only 1.3% of all bone marrow-related studies (Table 1). As a blood organ, bone marrow has been well established as the key niche for hematopoietic stem cells, which can regenerate the whole blood system from as little as one cell [49]. Concomitantly, bone marrow is also part of the bone organ as long bone, and its marrow almost always coexisst spatially and temporally. To this end, bone marrow has been postulated to be the niche for another stem cell type, the MSCs, although this matter is still under some dispute [50,51,52]. 

It is now unquestionable that bone marrow has its uses for bone regeneration. Early studies showed its osteogenic potential when fragments of bone marrow or bone marrow cell suspensions were implanted into various ectopic sites in animals, including subcutaneously, and resulted in de novo bone formation [53,54]. It was noted that even if the sample of bone marrow was small enough to avoid rapid necrosis, bone formation only occurred in the zone of outgrowing cells, implying that the marrow structure is incompatible with bone formation. Indeed, bone marrow therapy today is essentially a cell therapy. Kuznetsov et al. showed that populations of stromal fibroblast cells isolated from human bone marrow could differentiate into osteoblasts, and form bone when subcutaneously implanted into immunodeficient mice [55]. Interestingly, while new bone was formed by the human cells, new bone marrow was colonized by the host’s cells. Until now, a great number of in vivo studies, mostly in laboratory animals, support the use of bone marrow for bone healing and regeneration [56]. On the other hand, the evidence from clinical studies has as yet been insufficient to demonstrate significantly improved bone healing when using bone marrow aspirate or bone marrow stem cells for conditions such as long bone fractures [57]. Nonetheless, bone marrow is generally believed to improve the outcome of bone healing, which is usually ascribed to its high stem cell content. Indeed, the osteogenic potential of a bone marrow aspirate directly correlates to the number of osteogenic stem cells it contains [58]. This, in turn, depends on how the aspirate is harvested, which further contributes to the variable results reported in the literature [59]. To sum up, bone marrow tissue undoubtedly possesses one of the key components for successful bone regeneration: the stromal stem cells. This resource, however, is among the most difficult to be utilized, as will be discussed in the next section.

## 3. The Cells in Bone

Bone cells can be categorized into two lineages: the osteoblast lineage, representing the bone-forming axis (consisting of MSCs, pre-osteoblasts, mature osteoblasts, bone-lining cells, and osteocytes), and the osteoclast lineage, representing the bone-resorbing arm (consisting of macrophages, osteoclasts, and multinucleated giant cells, all derived from bone marrow haematopoietic stem cells). The balance between bone-forming and -resorbing is modified throughout life to attain and preserve skeletal size, shape, and structural integrity. 

Bone-forming cells are, understandably, the focus of bone tissue engineers. Mature osteoblasts are the only cells that can explicitly build bone by secreting bone matrix proteins and guiding mineralization. Depletion of mature osteoblasts results in an arrest of skeletal growth [60]. However, mature osteoblasts are short-lived; a subset is encapsulated within the newly-formed bone matrix, becoming osteocytes, while the others either undergo apoptosis or become inactive bone-lining cells [61]. During bone healing, osteoblasts are continuously replenished from the osteogenic cells, pre-osteoblasts and MSCs residing in such nearby bone compartments as the bone marrow, endosteum and periosteum [62]. In fact, MSCs are critical to the bone-healing process as the precursors of both osteoblasts (involved in intramembranous ossification) and chondrocytes (involved in endochondral ossification). Additionally, MSCs can secrete numerous trophic factors to establish and regulate a regenerative microenvironment, for which they have recently earned the epithet ‘in vivo drugstore’ [52]. Various regulatory pathways such as the Indian hedgehog (IHH), Notch, WNT, BMP, and FGF signaling pathways are involved in the differentiation of MSCs into osteoblasts or chondrocytes, although much remains unknown. There are also many questions related to MSCs in vivo that are still extant, foremost their origin [63,64]. 

MSC therapy for bone regeneration has gained enormous interest in the last decade thanks to the growing understanding of the role of bone stem cells in healing and the general methodological readiness for such experiments. MSCs have been isolated from various tissue sources (including bone marrow, periosteum, adipose tissue, dental pulp, and umbilical cord) and can be cultured in vitro to clinically-sufficient numbers (hundreds of millions of cells). They can even be primed to improve their in vivo survival and performance [65,66]. Importantly, clinical trials involving MSCs have demonstrated that such therapy is safe, with few detrimental effects [67]. Abundant proof-of-concept studies using MSCs in combination with various types of carriers and delivery methods have demonstrated efficacy for bone regeneration in different animal models [68,69,70,71]. However, many outcomes of the manipulation of MSCs for bone regeneration are inconclusive despite the increasing number of registered clinical trials [66,72,73]. One particular exception is the trials using allogeneic MSCs for the bone disease osteogenesis imperfecta (OI), also known as ‘brittle bone’ disease, in which the patients lack a functional type I collagen gene. Here, MSC therapy has been initially successful, especially given the severity and lack of an effective cure for the disease [74,75]. It is also worth noting that less than 10% of all MSC clinical trials are directly related to bone and cartilage disorders; the other 90% have been for cardiovascular disorders, autoimmune disease, liver disease, kidney disease, and skin disease, amongst others [66]. 

**Figure 2:**
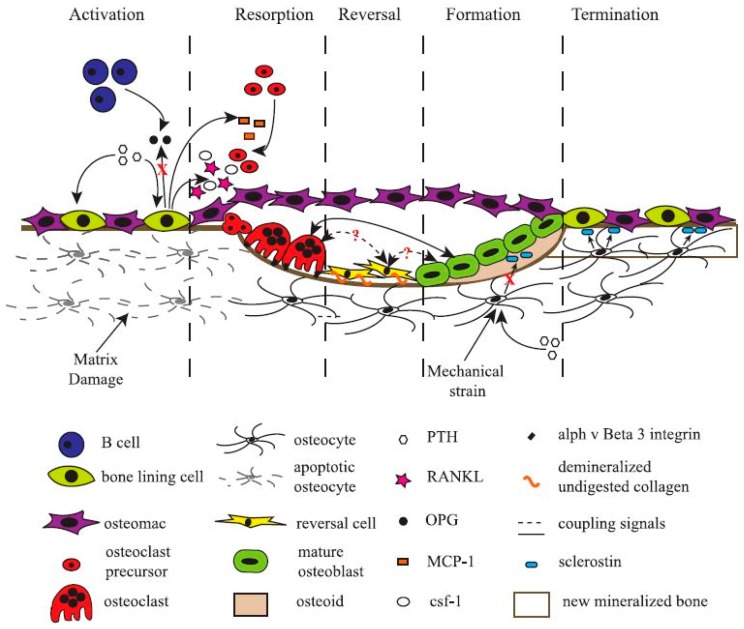
Schematic representation of a basic multicellular unit and the associated bone remodelling process. Permission obtained from ASBMB. © Liza J. R. and Nicola C.P. *J. Biol. Chem.* 285: 25103 (2010) [38].

More recently, scientists have turned their attention to another cell type found in bone tissue, the bone-resident macrophage, termed “osteomac” by Pettit et al. in 2013 [76,77,78]. Originally thought to only function as immune surveillance in the bone environment, osteomacs have recently been demonstrated to be indispensable for bone formation. Depletion of macrophages, and thereby osteomacs, results in a complete loss of mature osteoblasts and impaired bone formation [79,80,81]. Bone-forming cells only function in a specific micro-environment that is carefully laid down by osteoclasts, bone-lining cells, and osteomacs [38]. Osteomacs are the first cells to arrive at injury sites and interact with the foreign bodies [82]. They have been shown to form a distinct canopy-like cell structure that encapsulates functioning osteoblasts, thereby creating a specific working micro-environment for bone remodelling (Figure 2). Still, much remains unknown about the role, function, and interaction of every cell type in the bone-forming/-resorbing assembly. Bone cells, unmistakably the most valuable asset for any bone regeneration strategy, are paradoxically the most difficult to exploit.

## 4. The Extracellular Matrix of Bone

The extracellular matrix (ECM) holds the key to much of the naturally-occurring regeneration of a tissue or organ and, as such, tissue engineers have long sought to utilize natural ECM or ECM-mimicking materials [83,84,85]. There are different compositions of ECM associated with different parts of bone, including the periosteum, osseous tissue, and bone marrow. As osseous tissue best represents bone in terms of structure and function, its ECM is often considered a good starting material for its rebuilding. Osseous ECM is an interstitial matrix consisting mainly of an organic matrix (collagens, non-collagenous proteins, proteoglycans, and glycosaminoglycans) that bind tightly to hydroxyapatite (the mineral component). Whole osseous ECM, yet to be properly characterised, is made by processing bone tissue from allogeneic or xenogeneic sources (there is no reason to harvest and process autologous bone only for the ECM). Importantly, it is not that being derived from natural bone ECM makes for a good niche for bone cells. Indeed, no cell is able to squeeze through narrow canaliculi in order to re-inhabit the vacant bone lacunae. Therefore, any bone grafting material must be capable of being fully resorbed and replaced by new bone before the remodelling process completes [86]. Processing methods have been developed to make natural bone ECM more resorbable, including by physically breaking the tissue into smaller sizes (pulverization), chemically removing the mineral component (demineralization), or by partially enzymatically digesting the tissue (collagenase digestion) [87,88]. Alternatively, one can synthesize materials utilizing one or more of the constituent bone ECM components, of which collagen, calcium phosphate, and glycosaminoglycans are among the most often used. 

Type I collagen is the most abundant organic component of bone ECM, though it is not specific to bone, and is found in other tissues such as skin and tendons. Importantly, collagen is highly conserved in all vertebrates, and type I collagen from bovine and porcine sources has long been used in the clinic with good safety [89]. The role of collagen organization and its mineralization in the mechanical strength of bone is a complex subject and has been reviewed elsewhere [90]. As a bone substitute, however, collagen is the most extensively used biomaterial [91,92]. Physically, collagen materials can be made into injectable solutions, gels, malleable sponges, and solid forms of nearly any size and shape. Chemically, the collagen polypeptide possesses abundant functional groups, allowing modification, such as crosslinking to other biomolecules like glycosaminoglycans, RGD peptides, small molecules, and growth factors [93]. Overall, the collagen matrix induces haemostasis, improves biocompatibility of the synthetic scaffolds, and promotes tissue engraftment, all of which are important for any healing process [94,95]. Due to its ubiquitous presence in many different tissues, collagen is unlikely to promote any specific healing trajectory, but rather to create a stabilized microenvironment where the cells and their growth factor interactions determine the next phase of the healing process [96]. For critical-sized bone defects, collagen matrix alone has been shown to improve healing, but it is insufficient for complete bone regeneration [97,98]. On the other hand, the addition of BMP-2 on an absorbable collagen sponge can induce bone formation, even in ectopic locations [99]. 

**Figure 3:**
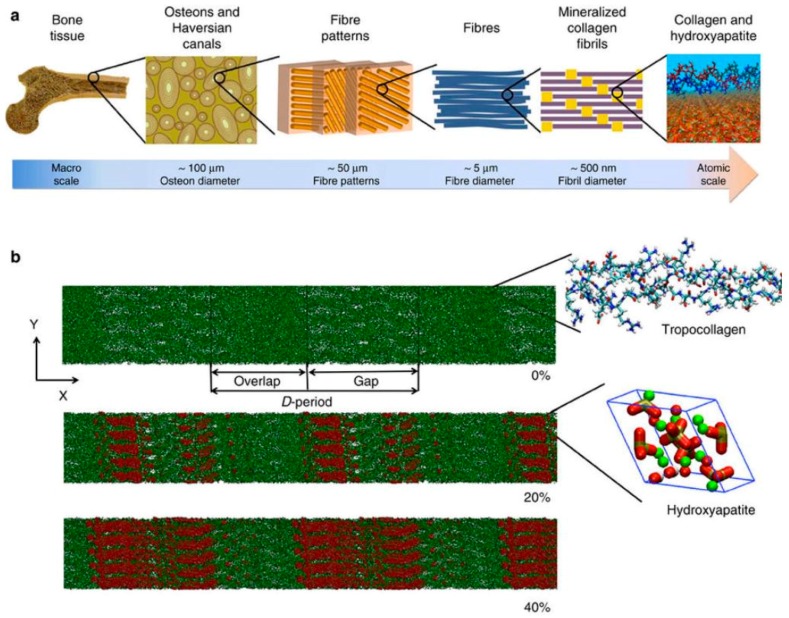
Organization of collagen and hydroxyapatite (HAP) in bone. (**a**) Hierarchical structure of bone ranging from the macroscale skeleton to nanoscale collagen (green) and HAP (red). (**b**) Collagen microfibril model with 0% mineralization (inset shows the collagen triple-helix structure), 20% mineral content (inset shows a HAP unit cell) and 40% mineral content. The HAP crystals are arranged such that the c axis of crystal aligns with the fibril axis. Ca atoms plotted in green, OH groups plotted in red and white, and the tetrahedron structure visualizes the PO4 group. Permission obtained from Springer Nature. © Nair, A.K. et al., *Nat Commun*. 4: p. 1724. (2013) [100].

The inorganic component of bone, the bone mineral (also termed bone salt or bone apatite), is a fascinating substance. Evolutionarily, as multicellular organisms became larger, possession of a hardened internal structure offered tremendous advantages. Through millions of years, nature has come up with a myriad of ways to create biomaterials that are not only hard, but also tough, light, and dynamic. Mineralization of collagenous matrix is the process that blends the inorganic mineral salts into a living matrix of cells and proteins, forming the bone composite. The chemistry of bone mineral primarily involves modifications of the calcium phosphate, hydroxyapatite (Ca_5_(PO_4_)_3_OH) [90,101]. What is unique is the way these molecules are put together in a specific manner to form a rigid, crystallized structure in tandem with the arrangement of collagen fibrils (Figure 3). This mineral has been researched since at least the 1770s [102]. Much is now known about its micro- and nanostructure, as well as its potential use for bone regeneration [103]. Calcium phosphate materials have been shown to not only be biocompatible, but, more importantly, they can be resorbed in the body without causing elevated calcium or phosphate levels in urine, serum, or other organs (liver, skin, brain, heart, kidney, lung, and intestine) [104]. Various types of calcium phosphate materials have been synthesized to differ in their chemistry or physical state, among them the group of ceramics, which have demonstrated superior performance for the support of bone regeneration [105]. Apart from having good osteoconductivity—attracting the host bone to grow—calcium phosphate ceramics have even demonstrated osteoinductivity—the induction of bone formation outside the bone environment [106,107]. The osteoinductivity of calcium phosphate ceramics is significant, considering this ability involves both chemoattraction and differentiation of stem cells into bone-forming cells at the material site. Although it is not clear exactly how this works, the bone-inducing mechanism of such ceramics has started to be elucidated. Factors, such as chemistry and topography, are being isolated and systematically studied [108,109,110,111]. Undoubtedly, calcium phosphate materials have been, and will be, used extensively in many bone regeneration strategies.

Glycosaminoglycans (GAGs) are essential building blocks for life. GAGs are ubiquitously present in all tissues, in the ECM, and on the surface of every cell. Unlike other biomolecules, such as nucleic acids and proteins, GAGs are produced as a result of non-template-driven synthesis and, thus, are highly heterogeneous. Of the four different groups of GAGs (heparin/heparan sulfate, chondroitin sulfate (CS)/dermatan sulfate, keratin sulfate (KS), and hyaluronic acid), CS and KS are the most abundant in bone [112,113,114,115]. Other than serving a structural role, GAGs, such as heparan sulfate (HS), are important modulators of cells and their interaction with growth factors [116,117]. The ability of HS to bind and protect growth factors from proteolytic degradation and even enhance the growth factor/receptor interactions has long been recognised. Many growth factors relevant to bone regeneration (such as FGFs, BMPs, TGFs, VEGFs, PDGFs) have been shown to bind to different species of HS through their unique heparin/HS-binding domains [118]. Unlike collagen, it is more difficult to isolate a large amount of HS from allogeneic or xenogeneic sources. The most convenient source is the by-product of anticoagulant heparin isolation from animal organs such as porcine intestinal mucosa [119]. For this reason, different approaches to chemically engineer molecules with HS-like properties have been attempted. 

One approach uses dextran derivatives (CMDBS), which mimic HS by protecting and stabilizing FGFs and have been shown to accelerate healing of cranial bones in a rodent model [120,121]. Indeed, a single dose of CMDBS delivered in a collagen sponge was shown to improve bone healing in critical-sized calvarial defects. As they later developed into a family of “regenerating agents”, these HS-like polymers were able to stimulate tissue repair in many different tissues including bone, skin, muscle and cornea [122] by mimicking HS binding to endogenous growth factors (released during the healing process) in a nonspecific manner, so stabilizing them for prolonged action. Jackson et al. took a different approach by isolating bone-derived HS from a culture of MC3T3-E1 pre-osteoblast cells that were used to augment fracture healing in rats [123]. The bone cell-derived HS enhanced growth factor activity within the callus, resulting in increased expression of osteoblastic genes. An even more specific approach was taken by Murali et al., using chromatography columns derivatized to capture HS species that have a high affinity for BMP-2 [124]. These affinity-isolated HS species enhanced BMP-2-induced osteogenesis overall by improving BMP-2 interaction with its receptor, thereby prolonging downstream signalling and, coincidentally, by also decoying the BMP-2 antagonist noggin. When the HS was combined with a collagen sponge to treat critical-sized bone defect in rabbits, the combination device was equally osteostimulatory to exogenous BMP-2.

A recent development in GAG engineering has been the discovery of supramolecular self-assembled amphiphile glycopeptides, which display a high aspect ratio charge on their surface; even higher than heparin, the most negatively-charged biomolecule in nature [125]. The glycopeptide amphiphile can bind strongly to many different growth factors, but, unlike heparin, it has almost no anticoagulant activity—the side effect that renders heparin inappropriate for tissue engineering applications. When combined with BMP-2 in a collagen sponge for a rat posterolateral spinal fusion model, the engineered sugar boosts the activity of a BMP-2 dose that is 100 times lower than the effective dose. GAGs, an often overlooked component of the bone ECM, should be more carefully considered in the design of future bone regeneration strategies.

## 5. The Future of Bone Tissue Engineering

The treatment of bone injury has been attempted as early as the beginning of medicine. Although not scientifically recorded, surgical interventions existed even in prehistoric times, as suggested by paleopathological evidence, such as set fractures, rickets, and drilled skulls [126]. Materials such as animal bone, ivory, silver, and gold have been used to replace missing teeth or bones. Later, as the result of the 19–20th-century technological revolution, the emergence of tools, such as high-resolution microscopy, histological and staining techniques, X-ray, computed tomography, and genomic and proteomic techniques, have boosted our understanding of bone biology enormously. Very large collaborative international research projects, such as the human genome project, the human proteome project, and the human protein atlas, have been completed or are underway. Rather counterintuitively, this increasing tide of information has not created a proportionally clearer understanding of the interactions between the key biological systems of the human body. Every new signaling pathway adds more complexity and, unlike electronics, no one-input-one-output mechanism exists in biology. For scientists looking for evidence for the best method to regenerate bone, it becomes clear that there is no best method—the right approach will depend on every individual biological situation. Thus, gaining in-depth knowledge about how every system in the body functions and malfunctions is a must before we can implement more perfect, personalized medical treatment. 

The second thing can be concluded from this recent influx of data is that any attempt to find a therapeutic solution using the usual trial-and-error approach will be increasingly unlikely to work. The mere number of possible therapeutic combinations exceeds the capacity of any high-throughput screening system. Fortunately, concomitant with the accumulation of “big data” is the rise of machine learning and other methods to replace or augment the human researcher. The important take-home message is: tissue engineering of bone (or any other living organs, for that matter) is not as simplistic as combining a few cell types on some scaffolds with certain growth factors, followed by implantation in vivo hoping for complete restoration of the tissue. As it moves from the bench to the bedside, a much deeper and more comprehensive understanding of bone biology, and medicine as a whole, must be applied to customize the most suitable bone regenerating therapy for an individual patient.

## Figures and Tables

**Table 1:** Summary of terms used in the PubMed search conducted in October 2017.

Tissue	Search Term	Number of Hits
Periosteum	Periosteum [Title] OR periosteal [Title]	2559
Osseous	Osseous [Title]	6091
Endosteum	Endosteum [Title] OR endosteal [Title]	446
Bone marrow	“bone marrow” [Title]	63,165
Bone marrow (related to bone research)	(“bone marrow” [Title]) AND ((“bone regeneration” [Title/Abstract]) OR (“bone healing” [Title/Abstract]) OR (“bone tissue engineering” [Title/Abstract]) OR (“fracture healing” [Title/Abstract]) OR (“nonunion” [Title/Abstract]) OR (“non-union” [Title/Abstract]))	805
